# Lemon Juice in Acute Articular Rheumatism

**Published:** 1851-10

**Authors:** 


*Lemon Juice in Acute Articular Rheumatism.*—The foreign journals contain fresh testimony in favor of the treatment of rheumatism by lemon juice. Dr. Perkins, of Brussels, and Dr. Giraud, of Grenoble, report in its favor in the *Journal des Connaissance Medico-Chirurgicales.*

